# The ReproGenomics Viewer: an integrative cross-species toolbox for the reproductive science community

**DOI:** 10.1093/nar/gkv345

**Published:** 2015-04-16

**Authors:** Thomas A. Darde, Olivier Sallou, Emmanuelle Becker, Bertrand Evrard, Cyril Monjeaud, Yvan Le Bras, Bernard Jégou, Olivier Collin, Antoine D. Rolland, Frédéric Chalmel

**Affiliations:** 1 Inserm U1085-Irset, Université de Rennes 1, F-35042 Rennes, France; 2 Institut de Recherche en Informatique et Systèmes Aléatoires (IRISA/INRIA) - GenOuest platform, Université de Rennes 1, F-35042 Rennes, France; 3 Ecole des Hautes Études en Santé Publique, Avenue du Professeur Léon-Bernard, F-35043 Rennes, France

## Abstract

We report the development of the ReproGenomics Viewer (RGV), a multi- and cross-species working environment for the visualization, mining and comparison of published omics data sets for the reproductive science community. The system currently embeds 15 published data sets related to gametogenesis from nine model organisms. Data sets have been curated and conveniently organized into broad categories including biological topics, technologies, species and publications. RGV's modular design for both organisms and genomic tools enables users to upload and compare their data with that from the data sets embedded in the system in a cross-species manner. The RGV is freely available at http://rgv.genouest.org.

## INTRODUCTION

Sexual reproduction in eukaryotes involves a wide spectrum of biological processes by which species give rise to new individuals and thus perpetuate. These include the formation of haploid gametes after meiosis, a specific type of cell division that takes place only in the germ line. In the male, the differentiation of germ cells into highly specialized spermatozoa is a complex and tightly regulated process called spermatogenesis. This developmental process involves the sequential and coordinated expression of thousands of genes, many of them testis-specific. Spermatogenesis has thus been widely explored by several microarray-based expression studies over the last two decades (1,2) and several databases devoted to spermatogenesis and gametogenesis (3–5) or to reproduction in general (6–8) have been developed to organize and provide access to this massive quantity of data.

More recently, ultra-high-throughput next-generation sequencing (NGS) projects have imposed new challenges on the life science research community: the complex tasks of processing, hosting and interpreting these data (9). The repositories or databases referred to above, however, cannot cope with several intrinsic features of NGS data. For instance, although microarrays provide an average measurement of gene or transcript expression that can be easily displayed, NGS offers quantification at a single-base resolution, a feature that could only be observed by specific visualization tools that can take into account both genome coordinates of sequenced nucleotides and coverage information along every genomic locus. Additionally, microarray-based expression databases are typically organized around annotated entities, i.e. probes, transcripts, genes or, perhaps, corresponding proteins. Their structure is therefore incompatible with the ability of RNA-sequencing to lead to new discoveries (e.g. when new transcript isoforms are assembled and/or new loci identified) and not adapted to ChIP- or Methyl-seq analyses of specific chromatin regions, the boundaries of which cannot be strictly defined. The so-called genome browsers, a new type of database, have emerged to meet these requirements (10). UCSC's famous website (11) is a pioneer in this regard. The implementation of new modules (12,13) makes it possible to create even more flexible and intuitive browsers. These allow the hosting, visualization, customization, retrieval and analysis of various types of genomics data in a single environment, thus enabling researchers to extract and share data easily and construct new hypotheses from them. Most of these browsers, however, focus on a single species (14–17) or a single type of genomic data (18,19). To our knowledge, there is no tool directed toward a specific research field and scientific community that can bring together the major relevant studies, regardless of species and technology type.

Here we present the ReproGenomics Viewer (RGV), a cross-species genomic toolbox for the reproductive community. The system is based on the implementation of a ‘JBrowse genome browser’ (20) and a ‘Galaxy bioinformatics workflow environment’ (21–23). It was developed to provide a one-stop genomic working environment and aims to assist scientists in the analysis and the mining of a wide range of high-throughput repro-genomics data, including sequencing data. RGV allows hosting, visualization and direct comparison of users’ data to published genomics studies as well as to relevant genetic variations linked to reproduction. One way it does this is by enabling various genomic file format conversions. These genomic coordinates can be converted not only between genome releases of a given species but also and more importantly between different species. This key feature allows the direct comparison of data sets acquired in different organisms and thus makes RGV not only a multispecies genome browser but also a true cross-species tool for comparing reproductive genomics data. The RGV currently hosts data sets that are oriented mainly toward testis biology and spermatogenesis. In the near future, these will extend to other areas of reproduction, including gonad development, urogenital cancers and reproductive toxicology.

## DESCRIPTION OF DATA SETS

As mentioned above, the RGV currently embeds 15 published studies related to male gamete development or gametogenesis in general (24–36) (Table 1). These data sets are publicly available through the NCBI Gene Expression Omnibus Repository (37). They describe the extensive re-exploration of the spermatogenesis process over the past few years by the emerging ultra-high-throughput sequencing technologies. Specifically, the studies investigated the dynamic omics landscape of developing male germ cells, including: (i) chromatin remodeling and epigenetic features such as active and repressive marks (24–25,27–30); (ii) cistromes of transcription factors important for spermatogenesis (26,29); (iii) transcriptional landscapes, defined mainly by RNA sequencing technologies (24,28,31–36); and (iv) proteomic profiles generated with the recent Proteomic Inferred by Transcriptomic approach (34). All these experiments took place in a wide spectrum of model organisms, including *Homo sapiens* (25,30,36), *Gorilla gorilla* (36), *Macaca mulatta* (36), *Mus musculus* (24–29,31–32,35), *Rattus norvegicus* (33,34), *Monodelphis domestica* (36), *Ornithorhynchus anatinus* (36), *Gallus gallus* (29,36) and *Saccharomyces cerevisiae* (as sporulation in yeast is the developmental process analogous to spermatogenesis in higher eukaryotes (38–41)). Taken together, these published data sets currently represent 342 samples, 168 of vertebrates.

**Table 1.** Published data sets relevant to gamete development currently included in the RGV system and some relevant characteristics

Publication	PubMed IDs	Species (release)	Technologies	Biological topics
Chocu *et al*., 2014 (34)	25210130	Rat (rn4)	RNA-seq	Spermatogenesis
Hammoud *et al*., 2014 (25)	24835570	Multi (2 species)	Chip-seq, Bisulfite-seq	Spermatogenesis
Chalmel *et al*., 2014 (33)	24740603	Rat (rn4)	RNA-seq	Spermatogenesis
Meikar *et al*., 2014 (35)	24554440	Mouse (mm9)	RNA-seq, smallRNA-seq	Spermatogenesis
Necsulea *et al*., 2014 (36)	24463510	Multi (7 species)	RNA-Seq	Tissue profiling
Soumillon *et al*., 2013 (32)	23791531	Mouse (mm9)	RNA-seq	Spermatogenesis
Erkek *et al*., 2013 (28)	23770822	Mouse (mm9)	RNA-seq	Spermatogenesis
Gan *et al*., 2013 (24)	23759713	Mouse (mm9)	RNA-seq, 5hMeDIP-seq	Spermatogenesis
Laiho *et al*., 2013 (31)	23613874	Mouse (mm9)	RNA-seq	Spermatogenesis
Li *et al*., 2013 (29)	23523368	Multi (2 species)	ChIP-seq	Spermatogenesis
Gaucher *et al*., 2012 (26)	22922464	Mouse (mm9)	RNA-seq	Spermatogenesis
Brick *et al*., 2012 (27)	22660327	Mouse (mm9)	ChIP-seq	Spermatogenesis
Lardenois *et al*., 2011 (38)	21149693	Yeast (sacCer3)	Tiling Array	Sporulation (SK1, MATa-alpha)
Brykczynska *et al*., 2010 (30)	20473313	Human (hg18)	MNase-seq	Spermatogenesis
Granovskaia *et al*., 2010 (41)	20193063	Yeast (sacCer3)	Tiling Array	Mitosis (W101, MATa)

In a critical step, we also gathered allele and genotype frequency data and significant genetic association findings from such public databases as GWAS and ClinVar (42,43). The control vocabulary provided by both projects enabled us to split genetic association studies into two categories: reproductive and non-reproductive symptoms. Direct links to PubMed and variant databases are provided.

## THE RGV BACKBONE: DATA PROCESSING AND ORGANIZATION

**Figure 1.**
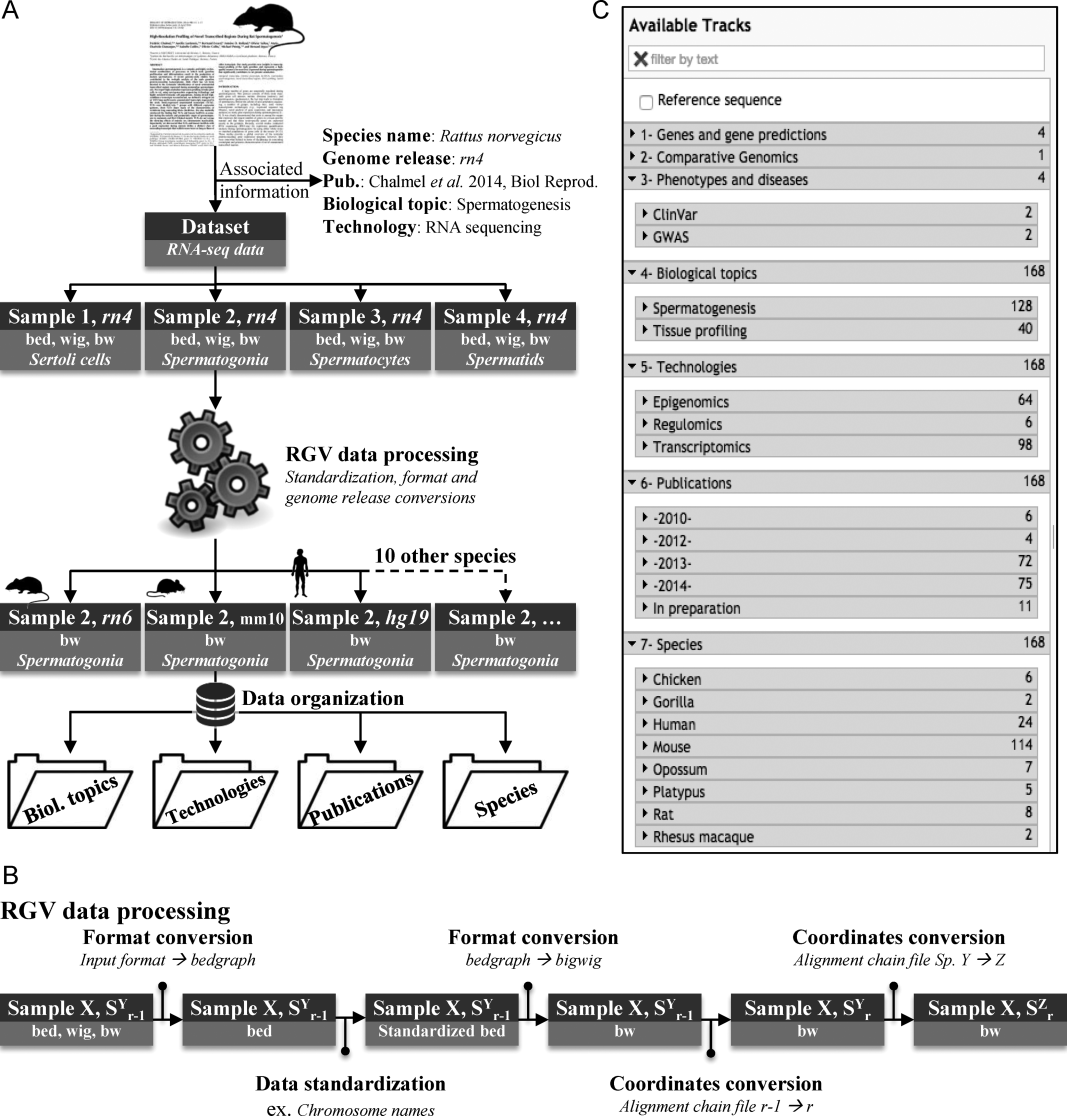
The RGV backbone. (**A**) A schematic diagram of the strategy used to process and organize each individual sample from the published data sets embedded in the RGV system. The publication by Chalmel *et al*. is taken as an example (33). The organization of the data is based on the information manually extracted from the publication (species name, genome release, biological topic and technology). (**B**) The ‘RGV data processing’ workflow used to convert data file formats, standardize data files and then to convert genome coordinates between assemblies (*r_−1_* → *r*) and between species (species *Y* → *Z*). (**C**) Screenshot of the JBrowse ‘Available tracks’ menu illustrating the ‘in-house’ organization of the published data sets embedded in the RGV system in several categories, such as ‘Biological topics’, ‘Technologies’, ‘Publications’ and ‘Species’.

The backbone of the RGV is the series of tools for processing and organizing data within the system (Figure 1A). Four types of information were manually extracted and curated for each study, including: the scientific name of each species and the genome release with which the experiments were performed and analyzed; the associated scientific publication; each biology topic investigated in the study; and the high-throughput technologies performed. Then each sample of a given data set underwent a series of automatic conversions to make it fully compatible with the RGV system (Figure 1B). Briefly, for a given sample *X* analyzed under a genome release *r-1* of Species *Y*, five processing steps were sequentially performed: (i) each of the various input data formats (bedGraph/BED, WIG, bigWig) was converted into a simple tab-delimited text file (BED); (ii) as some differences can occur even in the same genome release of a given species, the resulting BED data file might have needed to be modified to standardize, for example, the chromosome names that might differ between the Ensembl, UCSC and NCBI databases; (iii) the standardized BED file was then converted into an indexed binary format (bigWig or bw) to enable fast remote access to the data; the pairwise alignments between genome assemblies and between species provided by UCSC made it possible to convert genome coordinates in the resulting bigWig file from a genome release *r-1* of the species *Y* into (iv) the current assembly *r* of the same species *Y* and then (v) the current assembly *r* of another species *Z*.

Finally, we used manually extracted information to organize the processed data into four broad categories, i.e. biological topics, technologies, publications and species (Figure 1A). This organization is mirrored in the ‘Available Tracks’ option of the ‘JBrowse genome browser’ implemented in RGV (see the next section) to facilitate access to curated and relevant experimental data (Figure 1C).

## BIOINFORMATICS TOOLS DEPLOYED IN THE RGV WORKING ENVIRONMENT

The system also integrates an implementation of the ‘JBrowse genome browser’ (20) and of the ‘Galaxy bioinformatics workflow environment’ (21–23), grafted to the RGV backbone.

### The RGV working environment

To host genomics tools essential for data comparisons between genome releases and above all between species, we implemented a ‘Galaxy bioinformatics workflow environment’. Briefly, Galaxy is an open web-based platform for genomic research that provides users with an easy-to-use web interface to create complex biological workflows by tools that simply need to be dragged and dropped. It is worth mentioning that the ‘RGV Galaxy session’ is available without creating an account. Users are, however, strongly invited to create an account to have access to their history, saved analyses, data sets and workflows. By default, this environment contains a myriad of tools designed mainly to assist users in handling files; these are largely simple file manipulation tools to convert, filter, sort, select, extract features or combine files. The current release already uses this versatile Galaxy working environment to deploy two workflows.

The ‘RGV data processing’ workflow described in the RGV backbone section (Figure 1B) was conveniently implemented as a Galaxy module. This pipeline is based on the implementation of three tool suites: UCSC tools (44), bedtools (45) and CrossMap (46). The former is used for all data file format conversions in either bedGraph or bigWig formats. The second is employed for the data standardization step. Finally, the latter is used in both cross-assembly and cross-species conversions of genome coordinates and makes use of pair-wise alignment files (chain format) provided by UCSC. The entire process takes roughly 30 min for an input file (bam format) of 200 Mb. Once the conversion is completed, the user can easily upload the resulting bigWig file to the ‘RGV JBrowse session’.

‘A genome alignment workflow’ based on the Blast-Like Alignment Tool (BLAT) (47) was implemented as a tool in the Galaxy working environment. Briefly, it allows users to use a one-step procedure to automatically align their DNA/RNA or protein sequences (fasta format) onto the 13 reference genome sequences available in the RGV system. The resulting alignments are post-processed and made available in two forms: a table including direct links to the ‘JBrowse session’ and a General Feature Format (gff) file that can be uploaded to the genome browser.

### The RGV JBrowse session

As many more genomes, transcriptomes and epigenomes will be sequenced in the decade to come, a user-friendly genome browser has become essential for work in reproductive biology.

#### JBrowse advantages

The client-server architecture of JBrowse offers several advantages over other genome browser solutions, such as GBrowse (13): (i) the system is fully compatible with a wide spectrum of data types, including sequence files (fasta format), genomic feature files (gff), alignment files (bam) and quantitative data files (bedGraph, wig, bigWig); (ii) genome browsing is rapid even when multiple users are processing data simultaneously; (iii) JBrowse provides a user-friendly and highly flexible graphical interface in which users can efficiently pan and zoom over a genomic sequence region and turn genomics tracks on and off by simply clicking buttons.

#### Track organization

As mentioned above, RGV currently includes 15 published data sets. Each has been standardized and converted via the ‘RGV data processing’ pipeline. Data were then organized into four broad categories in the JBrowse track selector, from the information manually extracted from the original publications. These categories (Figure 1C) currently include: biological topics (spermatogenesis and tissue profiling), technologies (epigenomics, regulomics or transcriptomics), publications and species (nine species).

#### User interaction

The implementation of JBrowse allows users to download data sets embedded into the RGV genome browser by choosing a track of interest and then by clicking on ‘Save track data’. Users can also upload their own data sets (several file formats are allowed: gff3, gtf, bigWig, bam and vcf) in the ‘JBrowse session’ to compare them to the existing tracks by using the option ‘Open’ in the ‘File’ tab. If necessary the user can first run the ‘RGV data processing’ pipeline, implemented in the ‘Galaxy session’ (see the previous section), and then upload their own tracks into the system.

#### Example

During spermiogenesis, sperm chromatin is remodeled into a condensed inactive state due to the replacement of histones by protamines (48,49). The latter are small arginine-rich proteins binding DNA expressed in the late-stage spermatids of many animals and plants. We used the ‘RGV JBrowse session’ to illustrate the mammalian conserved expression pattern of the genes encoding PRM1, PRM2 and PRM3 which are clustered on the human chromosome 6 (Supplementary Figure S1). Once the genes have been selected with the search bar and the genome fixed to Human (hg19), three expression data sets from human, mouse and rat were compared (32–33,36). The corresponding tracks were accessed by (i) the ‘Publications’ tab, by selecting ‘2013>Soumillon *et al*.’, ‘2014>Necsulea *et al*.’ and ‘2014>Chalmel *et al*.’. Note that the ‘Available Tracks’ menu is organized so that the same tracks could have been identified by (ii) the ‘Technologies’ tab or by (iii) the ‘Biological topics’ tab. The examination of the displayed tracks highlights the specific post-meiotic expression of the genes encoding protamines, as well as its strong conservation across mammals.

## DISCOVERING NOVEL GENES ACTIVE IN SPERMATOGENESIS

The large variety of ultra-high-throughput data across many eukaryotic organisms encourages the use of the RGV as a testing ground for building novel scientific hypotheses on the basis of relevant, curated experimental data on reproduction. The possibilities are numerous, and the applications of RGV diverse. For example, the integration and visualization of pertinent transcriptome data and genome-wide association studies related to reproductive symptoms in the ‘JBrowse session’ may help to elucidate the mechanisms through which genetic mutations lead to reproductive disorders. Another example concerns the integration of active/repressive epigenetic marks and transcriptomic data, which may help to identify the role of specific epigenetic modifications in modulating the expression of genes involved in spermatogenesis.

**Figure 2.**
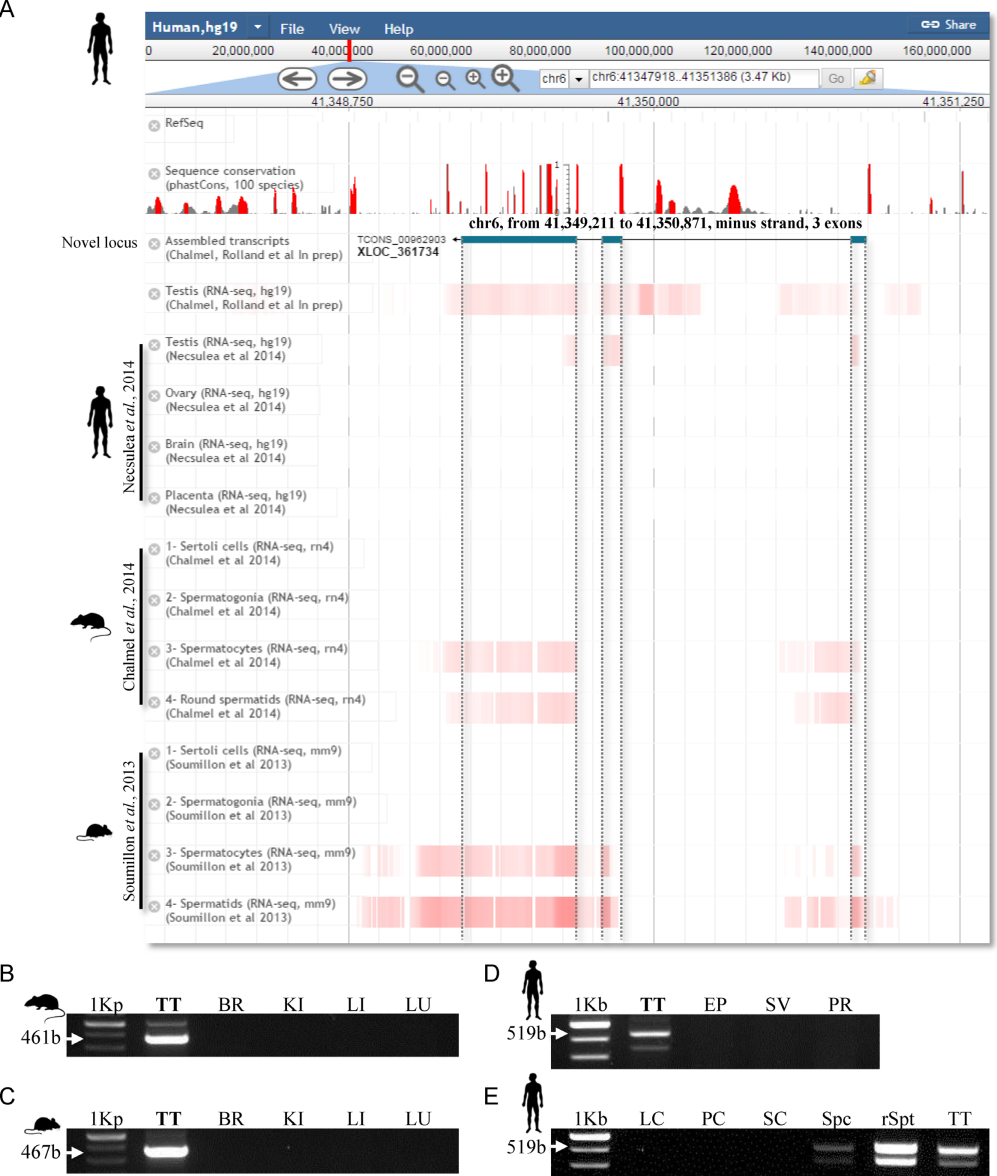
Tissue and cell-specific expression patterns of one novel intergenic locus are shown. (**A**) Structure of the novel intergenic locus (blue boxes correspond to introns), TCONS_00962903, in the human genome (release hg19), is displayed in the ‘RGV JBrowse session’. Four RNA-seq data sets were selected to illustrate the transcript abundance of this promising candidate in human testes (Chalmel, F. and Rolland, A.D., in preparation) (36) as well as in rodent meiotic and post-meiotic germ cells (32,33). The amount of transcript determined in each tissue/cell and in each study is displayed as color-coded red heat maps. Red histogram bars represent the sequence conservation score distributions between 100 species as provided by the UCSC genome browser (phastCons scores, y-axis ranges from 0 to 1). TCONS_00962903 detection at the RNA level was further confirmed by RT-PCR in four rat (**B**) and mouse (**C**) tissue samples, including total testis (TT), brain (BR), kidney (KI), liver (LI) and lung (LU). RT-PCR analysis was also performed in four human tissue samples (**D**), including total testis (TT), epididymis (EP), seminal vesicle (SV) and prostate (PR), as well as five isolated testicular cell populations (**E**) including Leydig cells (LC), peritubular myoid cells (PC), Sertoli cells (SC), spermatocytes (Spc), round spermatids (rSpt) and total testis (TT) as positive control.

To corroborate RGV's usefulness, we decided to test its ability to identify novel human loci dynamically expressed during male gamete development and conserved across species. We first integrated three RNA-sequencing studies in the ‘JBrowse session’: a tissue profiling project including samples from human testis and three other tissues (ovary, brain and placenta) published by Necsulea *et al*. (36); then we added two high-resolution expression profiles of male germ cells, one in rats (33) and the other in mice (32). Next, we analyzed the human testis sample provided by Necsulea *et al*. and assembled the transcripts with the cufflinks tool suite (50). We then sought to identify novel intergenic and multi-exonic loci that are expressed in human testes and have a meiotic and/or postmeiotic expression pattern in rodents (data not shown). This allowed us to select one promising candidate, designated TCONS_00962903, for further experimental validations to illustrate the relevance of our strategy (Figure 2A). This novel locus maps to chromosome 6 (positions 41 349 211–41 350 871) and is composed of three exons with a cumulative exon size of 659 bp. It shows preferential expression in testes compared with the other three tissue types in the study by Necsulea *et al*. (36). A simple examination of the ‘JBrowse session’, using the cross-species feature of RGV, showed very strong conservation in rodents, in which expression of this locus unambiguously peaked in spermatocytes and spermatids. This finding suggests its expression pattern in humans and rodents is similar (Figure 2A). Reverse transcriptase-polymerase chain reaction (RT-PCR) found substantial amounts of TCONS_00962903 RNA in human, mouse and rat testis samples, compared with the other tissue samples analyzed (brain, kidney, liver and lung for rodents; epididymis, seminal vesicle and prostate for humans) and thus confirmed its ‘testis-restricted’ expression pattern (Figure 2B–D) (Supplementary file S1). Finally, as suggested by the rodent RNA-seq data, we clearly confirmed that the expression of this novel gene in the testis is restricted to the human germ cells at spermatid stage (Figure 2E).

## FUTURE DEVELOPMENTS

In the near future we intend to extend the scope of the RGV to keep pace with rapid technological, bioinformatics/genomic and biological/clinical advances in the reproductive sciences. In concrete terms, we are currently planning four separate actions. First, we will gather other relevant data sets from a wide range of species in RGV to cover other reproductive biological topics (e.g. gonad development, oogenesis, reproductive cancers and reproductive toxicology). We have already selected 18 studies to integrate into the system (Supplementary Table S1), and we encourage data submission from colleagues. Second, we will be adding other genetic information related to reproductive disorders (such as GWAS and Quantitative trait loci information from diverse sources and diverse model organisms). Third, we plan to develop community tools that will greatly facilitate collaborative work and stimulate the emergence of novel forms of collaboration in our research field.

Finally, we will be enhancing the features and functionalities of the ‘RGV-Galaxy working environment’. In particular, we intend to embed the ‘JBrowse genome browser’ directly into the Galaxy environment. Users will thus be able to entirely customize, and eventually share, their own personal genome browser session with their ultra-high-throughput data sets. This integration of JBrowse within the RGV-Galaxy working environment will also facilitate communications and data export between the two sessions. Another crucial point involves the direct implementation of several workflows for analysis of NGS data (e.g. RNA-seq, ChIP-seq) within the Galaxy environment. This will have several user benefits, for it will enable reproductive biologists/clinicians to perform their own analyses independently. Above all, it will help to standardize data analysis procedures within the reproductive science community to facilitate comparisons of data sets.

## CONCLUSIONS

We report the development of the RGV, a webserver-based toolbox for reproductive scientists. The system combines specific solutions for ultra-high-throughput data management, curation and organization, with data conversion across releases and species (CrossMap), genome browsing (JBrowse session) and a bioinformatics workflow environment to deploy analysis pipelines (Galaxy session). RGV currently embeds 15 published data sets related to germ cell development from nine eukaryotic species. We intend to complete RGV's repertoire with other related biological processes, other model organisms and other technologies of interest related to reproductive biology in the near future. This may help scientists and clinicians who work on reproduction to compare their own data sets to relevant published studies in their specific field by overcoming the standard technical problems we face daily regarding data format, genome release and species issues. To the best of our knowledge, the RGV is the first cross-species working environment dedicated to a single biological field of interest. This community-based system could thus be applicable to other conserved biological processes studied in several model organisms.

## SUPPLEMENTARY DATA

Supplementary Data are available at NAR Online.

SUPPLEMENTARY DATA
