# Supplementary material for: The ReproGenomics Viewer: an integrative cross-species toolbox for the reproductive science community
Source: Nucleic Acids Res. 2015 Apr 16;43(Web Server issue):W109–16. doi: 10.1093/nar/gkv345 (PMC4489245; doi:10.1093/nar/gkv345)
Supplement: SUPPLEMENTARY DATA [file supp_43_W1_W109__index.html]

The ReproGenomics Viewer: an integrative cross-species toolbox for the reproductive science community — SUPPLEMENTARY DATA 

# The ReproGenomics Viewer: an integrative cross-species toolbox for the reproductive science community

## SUPPLEMENTARY DATA

**Files in this Data Supplement:**

- SUPPLEMENTARY DATA
- SUPPLEMENTARY DATA
- SUPPLEMENTARY DATA
