# Supplementary material for: The ReproGenomics Viewer: an integrative cross-species toolbox for the reproductive science community
Source: Nucleic Acids Res. 2015 Apr 16;43(Web Server issue):W109–16. doi: 10.1093/nar/gkv345 (PMC4489245; doi:10.1093/nar/gkv345)
Supplement: SUPPLEMENTARY DATA [file supp_gkv345_nar-00272-web-b-2015-File004.pdf]

Animal, human samples and ethical considerations

Adult male Sprague-Dawley rats and male C57Bl/6 mice were used for tissue collection (testis, brain, kidney, liver and lung) and RT-PCR experiments. They were purchased from Elevage Janvier (Le Genest-Saint-Isle, France). Animal experiments were performed in conformity with the principles for the use and care of laboratory animals and in compliance with French and European regulations on animal welfare.

Human materials were obtained at Rennes University Hospital from patients: normal testis and epididymis samples were collected at autopsy; normal seminal vesicles were collected from patients who underwent radical prostatectomy; prostate tissues were obtained from healthy men who underwent prostatic adenomectomy for benign prostatic hyperplasia. The local Ethics Committee approved the study protocol, "Study of normal and pathological human spermatogenesis", registered under n° PFS09-015 at the French Biomedicine Agency. Human tissues used for RT-PCR were frozen in liquid nitrogen and stored at −80°C until analysis. Human testicular cells (spermatids, spermatocytes, Sertoli cells, peritubular myoid cells, and Leydig cells) were isolated from human testes as described in (Chalmel *et al.*, 2007) (Chalmel, Rolland *et al.*, in prep.).

RNA isolation and RT-PCR

RNA was isolated using the RNeasy miniextraction kit (Qiagen) and treated with DNase (Promega). Complementary DNA was obtained from 500 ng of total RNA using the High capacity RNA-to-cDNA Kit (Applied Biosystem). Conventional PCR was performed using *Taq* polymerase (Qiagen) and a Peltier thermocycler (Labgene). Forward and reverse primers are listed below (overlined in yellow) for human, mice and rat TCONS respectively. Sequences and product sizes (overlined in blue) are indicated.

Human

```
GGGACAGGAGCCCTAGGCAATATGTATTAAGTAGACTTTCATGAACTCAACGAGATACTGCAAGAGATAAAATAA
TGAGATGCTACGTCATAGGATCTGTCTCTGAGTCCACATTGTGGGAGAAAAGACCATTCTTCGGAAAGATGCAGA
GGAAGCCCCGTACCTCAAGGGATTGAGATGGGAGACTGAGGGAATCCTGGGGTCTCTGCCCTCTCCCTCACTGGG
AAACAAGCTCTGTTTCTTCATGTGCTGGAGCCCTGCCTCTGTGGGTGCAGCCTGAGGACAAAGGCAGTGCTGCAT
CCCCTGATCCCCTGCCCCCTACAACCTCCTGCCCAGCCTCTTGGGTGCTCCTCAGCTTGTCGTCAGGGAGGAAGC
AGCCATCTCCTCCGATGACTGACTTCAAGCCTCAACTCTCCCACAGCTTCTCAAAAGCCAAGGCTGAGGCCAAGT
CCTAGCTGCTGCCTTGGGACAGGTCAAGGGCCTCCTGCAATCTCATGCACAAA CTGGTACCCATGAGCATGTGGC
CTAAGAGATTTATTGACCCCTCTCCAAGTCCTTGCCAGAGGAATCCTGCACACCTATATCCACAATGAAGGGAGC
CATATCCCCAGCTGGGTAGCCATA
```

Forward primer: CAGGAGCCCTAGGCAATATG
Reverse primer: CACATGCTCATGGGTACCAG

SEQUENCE SIZE: 624
PRODUCT SIZE: 519

Mouse

```
GACATCACAGCAGGGAGAGGGGCCCTTCACAACCTTTCCCACTTGGACTTTCAAGGACTCAGAAAGCTAGTGCTA
GAGAAAGAAGATCATGTCCTTGGGAAAGATGGAGAATGAGTTCGAAGTCAGGAAAGCCCGACTCTACAAGGGACT
GAGATGGAAGACCCAGACCCAGGGCGACCCGCACTCCCACTCACCTGGGAATGAGCTCTGCCCCTCCACGGAGCA
AGCTTCAAAGGTCTGCCTGTGTGGCTGCAGCTTGCGGACAAAAGCGGTCCTCCACCCCAGGCCGGATCCAGTCCC
TCTTCTAGTCTCTCGGGGGCCGTCCAGCTTGCCATCAGAGAGGAAGACACTGGTTTCCAAGGAGACAGTCTTTCC
AGCTCAGCTCTCCCACCACTCTCCGAAGGAAAAAGCTGAGGGCAAGTCCCAGCTGTTATCTTGGCGGGATCCGGT
ACCTCTGCGCCAGCTGGTGCCTATGAGCGTGTGGCCCAAGGGCTTTATTCATCCACTCCCCAAACCTTTGCCAGG
```

GCAAGCTCTGAGCACCTTTATCCATGTTGAAGGGGTTCACATCCCTAGGGCAGTATCTCCGGTGACATCACTGGT
GGAAGATAACAAGTCTTCTATGCCACCTTTACTAAAATGAACGTGCCTGCTCAATTAATTGCTATTTATCTGTGT
CTACTGTAAACACGCAAAACTGTCCATTTTTAACTGAGAATATATTTTTATCCTCAATGTGAACCCAGTTGGGAC
TTCCAGAATACTGTATCCCAGTGTATCTTTCAAAATATATTTTTATTTTTATTTGTGCATGT

Forward primer: GAGGGGCCCTTCACAACCTTT
Reverse primer: CACACGCTCATAGGCACCAG

SEQUENCE SIZE: 812
PRODUCT SIZE: 467

Rat

GACATCACAGAAGGGGAGGGGCCCTTCACAACTTGTCCCGCTTGGACTTTCAAGGACTCAGAAAGCTAGTGCTAG
AGAAAGAAGAGCATGTCCTTGGGAAAAATGGAGGATGATTTCGAAGTCAGGAAAGCCCGACTTTACAAGGGGTTG
AGGTGGAAGACCCAGACCCAGGACGTCCAGCACTCCCACTCACCTGGGAATGAGCTCTGCCCCTCCACGGAACAC
GCTTCAAAGGTCTGCCTGTGTGGGTGCAGCTTGCGGACAAAAGTGGTCCTCCATCCCAGGCCGGACCCACTTCCG
GCCCCTCGGGTGCCTCCCAGCTTACCATCGGAGAGGAAGACACTGGTTTCCAGGGATACAGTTTTTCCCGTTCAG
CTCTCGAATGACTCATTGAAGGAAAAAGCTGAGGGTAAGCCCCAGCTGTTACCTTGGCGTGACCCGGTACCTCTG
CGCCAGCTGGTGCCCATGAGCGTGTGGCCCAAGGGCTTTATTGACCCGCTCCCCAAGCTTTTGCCAGAGAAAGCT
TTGAGCACCCTTATCCATATTGAAGGGATTCACATCCCCAGGGCAGTATCCCCCGTGATGGCACTGGGGAAAGAT
ACCAAATCCTCTCTGCCACCTTTACTGAAATGAGCGTGTCCACTCAATTAATCACTAATTTTCTGTATCTACTGT
AAACATATGAAGCTATTTTAATGGAGAATATATTTATATTCTCATTATGACCCCAGTGGGGACTTCCAGAACACT
GTATCCCAATGTATCTTTCAAAATACATTT

Forward primer: GAGGGGCCCTTCACAACTTG
Reverse primer: CACACGCTCATGGGCACCAG

SEQUENCE SIZE: 780
PRODUCT SIZE: 461
