# Supplementary material for: The ReproGenomics Viewer: an integrative cross-species toolbox for the reproductive science community
Source: Nucleic Acids Res. 2015 Apr 16;43(Web Server issue):W109–16. doi: 10.1093/nar/gkv345 (PMC4489245; doi:10.1093/nar/gkv345)
Supplement: SUPPLEMENTARY DATA [file supp_gkv345_nar-00272-web-b-2015-File005.pdf]

**Supplementary Table S1 - Published datasets relevant for the reproductive science community selected for integration within the RGV system.**

| Publications | Pubmed IDs | Species (release) |
|---|---|---|
| Zimmermann et al. | Submitted | Mouse (mm9) |
| Chalmel, Rolland et al. | In prep. | Human (hg19) |
| Becker et al. | In prep. | Yeast, sacCer3 |
| Mu et al., 2014 | 25228648 | Mouse (mm9) |
| Wang et al., 2014 | 24813617 | Mouse (mm10) |
| Margolin et al., 2014 | 24438502 | Mouse (mm9) |
| Smagulova et al., 2013 | 23870400 | Mouse (mm9) |
| Jiang et al., 2013 | 23663777 | Zebrafish (danRer7) |
| Yokobayashi et al., 2013 | 23486062 | Mouse (mm9) |
| Ng et al., 2013 | 23352811 | Mouse (mm8) |
| Shen et al., 2012 | 23235881 | Mouse (mm9) |
| Sleutels et al., 2012 | 22709888 | Mouse (mm9) |
| Khil et al., 2012 | 22367190 | Mouse (mm9) |
| Lavigne et al., 2012 | 21997732 | Yeast (sacCer3) |
| Tan et al., 2011 | 21925322 | Mouse (mm9) |
| Smagulova et al., 2011 | 21460839 | Mouse (mm9) |
| Bernstein et al., 2010 | 20944595 | Human (hg19) |
| Xu et al., 2009 | 19169243 | Yeast, sacCer3 |
