# Supplementary figures and images for: The ReproGenomics Viewer: an integrative cross-species toolbox for the reproductive science community
Source: Nucleic Acids Res. 2015 Apr 16;43(Web Server issue):W109–16. doi: 10.1093/nar/gkv345 (PMC4489245; doi:10.1093/nar/gkv345)

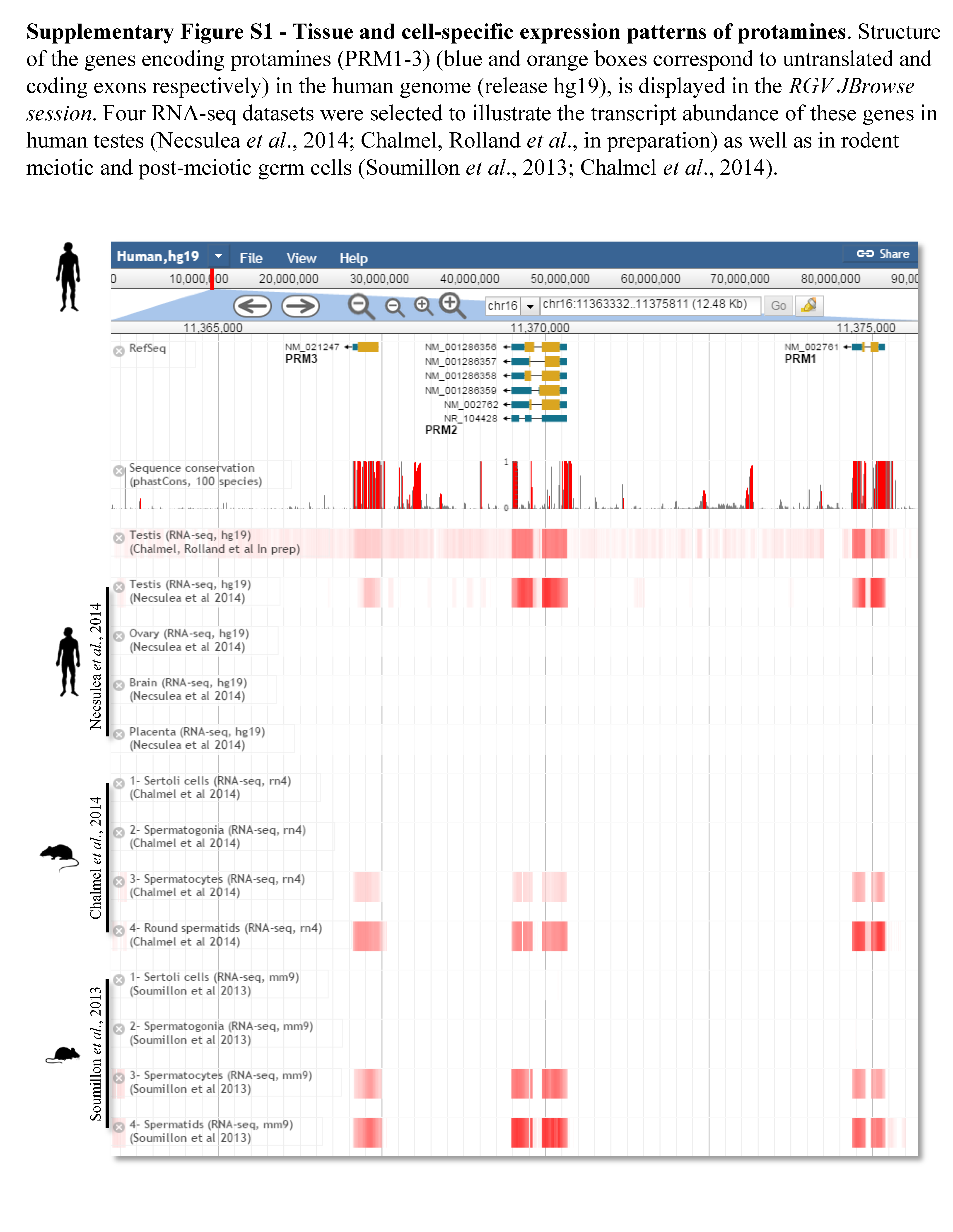

Supplement: SUPPLEMENTARY DATA [file supp_gkv345_nar-00272-web-b-2015-File006.png]
